# Editorial: Green Veterinary Pharmacology and Toxicology: a “One Health” Approach milestone

**DOI:** 10.3389/fvets.2024.1476877

**Published:** 2024-09-09

**Authors:** Cristina Carresi, Marianna Pauletto, Enrico Fiore, Vincenzo Musolino, Domenico Britti

**Affiliations:** ^1^Veterinary Pharmacology Laboratory, Department of Health Sciences, Institute of Research for Food Safety and Health IRC-FSH, University Magna Graecia of Catanzaro, Catanzaro, Italy; ^2^Department of Comparative Biomedicine and Food Science (BCA), University of Padova, Padova, Italy; ^3^Department of Animal Medicine, Production and Health, University of Padova, Padova, Italy; ^4^Pharmaceutical Biology Laboratory, Department of Health Sciences, Institute of Research for Food Safety and Health (IRC-FSH), University Magna Græcia of Catanzaro, Catanzaro, Italy; ^5^Department of Health Sciences, University of Catanzaro Magna Græcia, Catanzaro, Italy; ^6^Interdepartmental Center Veterinary Service for Human and Animal Health, University of Catanzaro Magna Græcia, CISVetSUA, Catanzaro, Italy

**Keywords:** Green Veterinary Pharmacology, One Health (OH)—Approach, antimicrobial resistance (AMR), phytocomplexes, plants, natural extracts

To date, climate change, the phenomenon of anthropization, and the widespread presence of environmental pollutants have significantly impacted the environment globally. These factors affect the wellbeing of animals and, through the food chain, also interfere with human health.

Furthermore, since the beginning of the antimicrobial era, the use and abuse of antibiotics in intensive farming have led to the onset of antimicrobial resistance (AMR) which represents a complex and unpredictable phenomenon requiring careful management (1, 2).

The long-term consequences of these profound changes are of growing concern; hence, setting novel scientific approaches envisaging environmental health, animal wellbeing, and consequently food safety and public health, as closely interconnected topics, is urgent and mandatory.

In this scenario, Green Veterinary Pharmacology (GVP) is a complementary and sustainable strategy to reduce the use of chemicals and minimize the phenomena of drug resistance and the persistence of residues in the environment, in a One Health Approach.

Recently, GVP approaches have been successfully applied due to their significant antibacterial activity (3, 4), efficacy in parasite control in poultry (5), small ruminants (6, 7), and bee farming (8, 9). In these studies, the identification and characterization of molecules of natural origin with anthelmintic properties have provided valid alternatives to canonical pesticides, thus limiting their environmental impact, soil and groundwater pollution, and the resulting long-term toxicity in treated animals.

Furthermore, the identification and mode of action of phytocomplexes can be useful in nutritional, pharmacological, and therapeutic applications for pets and farm animals.

Animal health is indisputable, and it affects the control of infectious diseases, and the quality and duration of life. In turn, it has a critical impact on the production and safety of animal-derived food products. Hence, it would be of great interest and auspicious that this approach finds fertile ground in veterinary medicine.

This Research Topic has collected eight interesting contributions covering the above-mentioned aspects.

In the work of Berny et al., the pharmacokinetic of fluralaner, a current external ectoparasiticide belonging to the class of isoxazoline, was evaluated in captive wild carnivores. The authors demonstrated that most carnivores excrete fluralaner in the feces for several weeks to months and that its duration of action can be quite variable between species: lions have a much longer elimination half-life than other smaller species where fluralaner rapidly disappears.

Despite some limitations of this study, mainly due to the constraints of working on wild animals in captivity, this research warns against the long-term excretion and potential persistence of fluralaner residues in feces and soil, that might result in non-target poisoning of various insect species, including dung beetles and flies (10).

In addiction, Dai et al., highlights how antibiotics concentration on the microbial community of water and sediment decreases the richness and diversity of microbial community. At a higher enrofloxacin concentration, with the prolongation of action time, the richness and diversity of bacterial community showed a downward trend and then gradually rose. The higher the concentration of enrofloxacin added, the greater the impact on the community and structure of microorganisms in the aquatic environment, and the number of drug-resistant bacteria will increase (Dai et al.).

Given this and the extensive scientific evidence available in the literature, the worldwide need to work on complementary and sustainable strategies to reduce the use of chemical substances and antibiotics to minimize the phenomena of drug resistance and residues persistence in the environment appears evident.

In this viewpoint, Li et al. in their review brightly examined possible alternatives to the use of antibiotics in bovine mastitis, a worldwide impactful disease due to its high prevalence, development of AMR and associated economic losses (11).

The authors underlined clinical effects, efficacy, and possible applications of non-steroidal anti-inflammatory drugs (NSAIDs) and bacteriophages in treating mastitis. For example, they reported the significant effects of meloxicam and carprofen, selective inhibitors of COX-2, which greatly reduce the incidence of mild to moderate mastitis, increasing feed intake and milk production, and reducing udder swelling and systemic inflammation (12, 13).

They hypothesize that NSAIDs may replace antibiotics in the treatment of mastitis in the absence of bacterial growth or in most infections with Gram-negative bacteria, avoiding the emergence of resistant strains since the mechanism of action of NSAIDs is not anti-bacterial.

According to the author's reflection, bacteriophages, in particular lytic phages, represent the most prospective successor to antibiotics for bovine mastitis. The authors mentioned numerous studies conducted to isolate different phage strains from milk and teat skin of cows with mastitis or from environmental sewage and test their antibacterial efficacy, alone or in mixture, with different receptors and complementary hosts to avoid bacterial resistance (14, 15).

On the other hand, herbal medicines, antimicrobial peptides (AMPs), and vaccination are reported to be useful in the prevention as they regulate the immune system (Li et al.). Indeed, compounds of natural origin and their bioactive metabolites represent another important alternative to the use of antibiotics in the prevention and treatment of bovine mastitis, as they exhibit a mechanism of action like that of antibiotics, but without or with minimal amount of side effects and residues in the milk (16).

The potential beneficial properties of several herbal medicines and their extracts, including *Red ginger, curcumin, maize whiskers, Terminalia Chebula*, are fully described in Xiaoping's review. As reported, these compounds show a good bactericidal effect against various mastitis pathogens without stimulating drug resistance with prolonged use (17–19). In addition to their powerful antibacterial action, essential oils from black seed, chamomile, or oregano, can be used to improve feed efficiency, enhancing nutrient supplementation, immune activity, and therefore animal health (20).

The advantages of herbal medicines are undeniable but, to date, few herbal drugs have been approved by the FDA for clinical use, probably due to the complexity of their composition, suggesting more accurate planning in the efficacy and safety evaluation phase (21).

Likewise, the naturally occurring antibiotic-like molecules (AMPs) are key components of innate immunity (22) for which excellent broad-spectrum antimicrobial activity has been documented (23, 24). Nevertheless, the application of AMPs requires further investigation considering the reported potential cross-resistance between AMPs and conventional antibiotics (25, 26). Similarly, there are still many difficulties in using phages as first-line agents, due to the absence of adequate efficacy and safety tests (27).

The authors also report interesting data regarding the potential of vaccines in the prevention of mastitis in cows. Vaccines, particularly those developed using recombinant protein technology, have great potential in reducing the incidence of mastitis, thus effectively reducing the use of antibiotics (28). However, the wide range of mastitis-causing organisms, especially environmental pathogens, far exceed the bacteria targeted by existing vaccines posing challenges to the development of new effective vaccines.

Other antibiotic-free strategies for treating bovine mastitis are currently under investigation. For example, photodynamic therapy, that alters bacteria cell membranes and DNA by inducing ROS production (29), or devices that use low-power acoustic (ATP) pulses to penetrate deep tissue and disperse pressure waves over a large region of the udder are promising approaches (30).

In addition to mastitis, two other livestock diseases that require extensive use of synthetic drugs, against which drug-resistance phenomena have been frequently reported, are endoparasites and coccidiosis. The study by Štrbac et al. is a brilliant example of how a botanical extract might be a valuable green alternative for the control of gastrointestinal nematodes (GINs) in sheep. In this study, peppermint *Mentha x piperita* essential oil (EO), chemically characterized by GC-MS, showed *in vitro* (egg hatch test) and *in vivo* (fecal egg count reduction test) anthelmintic efficacy. In particular, in the *in vivo* trial, *M. piperita* EO demonstrated a mean efficacy of 46.04% at day 14, in the absence of clinical side effects and impairment of hematological parameters. Despite some differences in efficacy observed between the two farms included in the trial, due to specific animal husbandry, Štrbac et al. demonstrated that *M. piperita* EO could represent a valuable environmentally friendly alternative to commercial drugs in the control of GINs.

EOs might represent a promising GVP also in tackling coccidia infections, as reported in the study by Han et al.. In this study, the efficacy of two EOs and six plant extracts, and their mixture, against *Eimeria tenella* was tested in chicken embryo fibroblast (DF-1). Non-toxic amounts of all the extracts and EOs significantly inhibited sporozoites invasion, being the mixture the most effective treatment. The efficacy of the mixture was then proved in 21-day old experimentally infected broilers through clinical and histopathological measures. In detail, the mixture showed improved feed conversion ratio and body weight gain, reduced fecal oocyst excretion and caecum damage, and increased survival. Overall, Han et al. provided the scientific community with striking evidence of the anticoccidial activity of a complex mixture of botanical extracts and EOs, thus being potentially employed for the prevention and control of *E. tenella* infection in the future.

Similarly, Castagna et al. identified an interesting anthelmintic activity of a Calabrian ethnoveterinary aqueous macerate based on *Punica granatum* (whole fruits) against GINs in Comisana pregnant sheep. Indeed, the fecal egg count reduction test showed a mean reduction of 52.5% after 21 days. Interestingly, a quantitative and qualitative examination of milk after anthelmintic treatment with the pomegranate mixture was conducted. The MilkoScan TM fT + foss electric (Hillerød, Denmark) recorded a higher milk production rate quantitatively (15.5%) and qualitatively (5.12% protein, 4.12% casein, 4.21% lactose, and 8.18% fat) in the pomegranate-treated group compared to the control group. The data obtained in this work contribute to strengthening the scope of GVP and provide possible green future approaches to counteract anthelmintic resistance in sheep farming.

The growing attention to the use of natural compounds in veterinary practice was, also, highlighted in the cross-sectional survey of Romero et al.. Through an online questionnaire carried out among Spanish small animal veterinarians, the authors reported widespread usage patterns and positive attitudes toward the use of herbal medicines (80.3%).

The main reasons for the use of phytotherapeutic drugs among veterinarians were identified in their benefits as adjuvant therapy (38.9%), and in the awareness of existing scientific evidence (34.1%). In most cases, phytocomplexes are used as therapy against digestive disorders and musculoskeletal and dermatological diseases as also reported in other studies (31, 32).

Interestingly, as reported by the authors, the lack of information and specific academic training were the main reasons why a small proportion of professionals included in the study did not use phytotherapy, suggesting the need for a broad discussion on the source of veterinarian's knowledge in this field and the lack of academic training provided from veterinary faculties.

Therefore, proper dissemination and specific academic training must go hand in hand with qualitatively as well as quantitatively advanced scientific research.

In this regard, a latest brilliant work conducted by Yin et al. greatly contributes to enriching the field of GVP from the point of view of scientific research. The authors studied the effective components and molecular mechanisms of Wumei San (WMS), a traditional Chinese medicine containing *Mume Fructus, Coptis chinensis* Franch., *Curcuma longa* L., *Terminalia chebula* Retz., and *Diospyros kaki* L.f., in treating piglet diarrhea (PD), using network pharmacology and molecular docking (Yin et al.). Briefly, the authors used molecular docking to examine the relationship between the main active compounds of WMS and the target proteins identifying a total of 32 active compounds and 638 target genes of WMS, constructing a WMS-compound-target network and successfully revealing that the active compounds in WMS spontaneously bind to their targets. Through network pharmacology analysis, 14 core compounds in WMS that showed an effect on PD were identified. Then the targets revealed by GO and KEGG enrichment analysis were associated with many signaling pathway, such as PI3K-Akt, TNF, NOD-like receptor IL-17 signaling pathway, and other physiological processes. These excellent results indicated that WMS may regulate the local immune response and inflammatory factors mainly through the TNF signaling pathway, IL-17 signaling pathway suggesting that WMS is a promising treatment strategy for PD.

In conclusion, considering the interesting contributions discussed, GVP and Toxicology represent a branch of veterinary pharmacology that should be implemented as a complementary and sustainable method to reduce the use of chemical substances and minimize drug resistance phenomena and the persistence of residues in the environment, promoting the study and use of compounds of natural origin derived from plants and natural products in a perspective of a “One Health” signal approach. In turn, the “One Health” initiative would help expand global collaborations across all aspects of healthcare for humans, animals, and the environment.
